# Supplementary material for: Genome-wide association study for circulating metabolic traits in 619,372 individuals
Source: medRxiv. 2025 Apr 12:2024.10.15.24315557. Preprint. [Version 3] doi: 10.1101/2024.10.15.24315557 (PMC12036396; doi:10.1101/2024.10.15.24315557)
Supplement: Supplement 2 [file NIHPP2024.10.15.24315557v3-supplement-2.pdf]

medRxiv preprint doi: https://doi.org/10.1101/2024.10.15.24315557; this version posted April 12, 2025. The copyright holder for this preprint (which was not certified by peer review) is the author/funder, who has granted medRxiv a license to display the preprint in perpetuity.
It is made available under a CC-BY 4.0 International license .

# Supplementary Figures

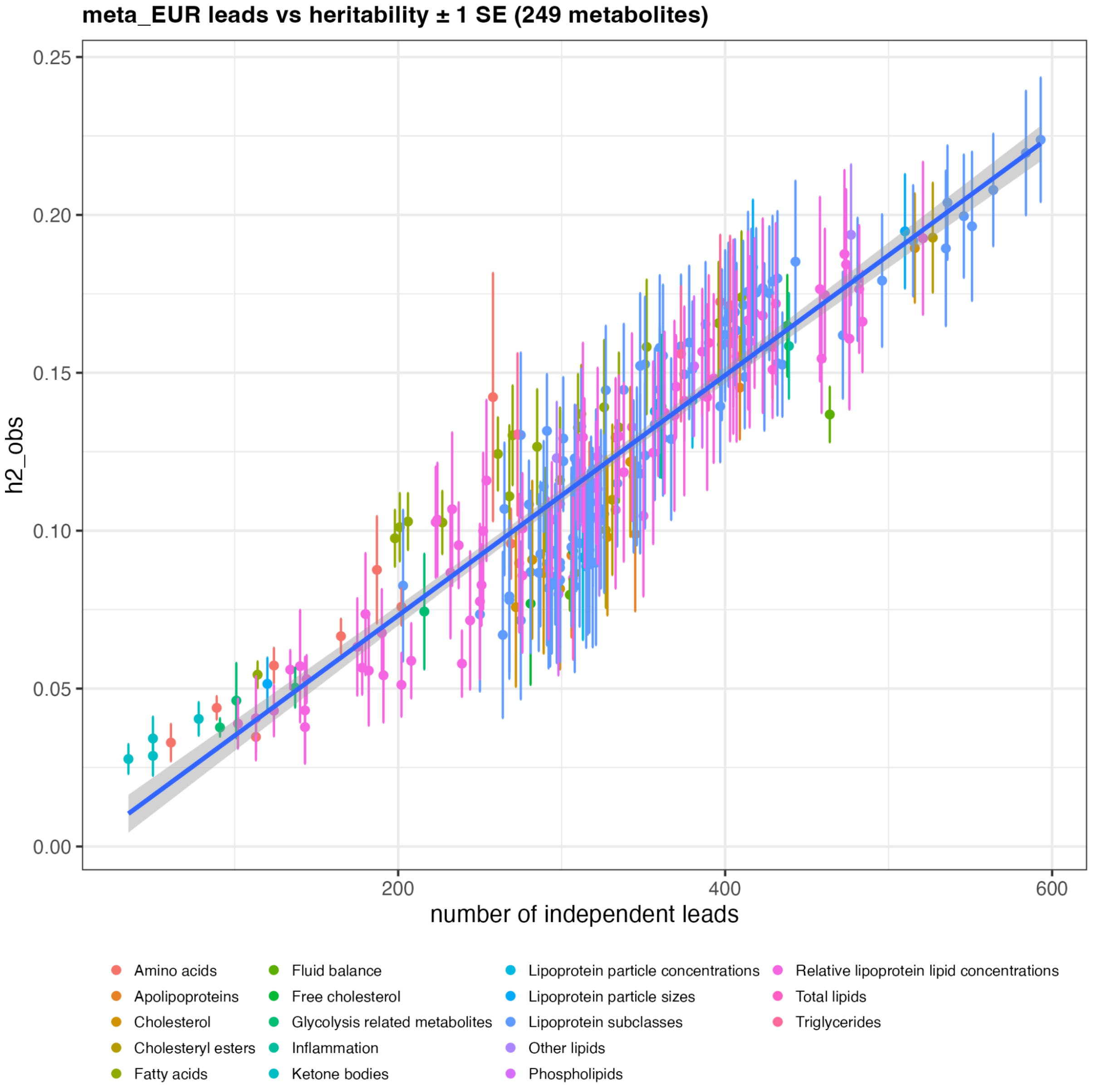


**Figure S1.** Relationship between heritability (h2_obs) and the number of genome-wide significant hits detected for each of the 249 metabolic traits in the meta_EUR meta-analysis.

medRxiv preprint doi: https://doi.org/10.1101/2024.10.15.24315557; this version posted April 12, 2025. The copyright holder for this preprint (which was not certified by peer review) is the author/funder, who has granted medRxiv a license to display the preprint in perpetuity.
It is made available under a CC-BY 4.0 International license .

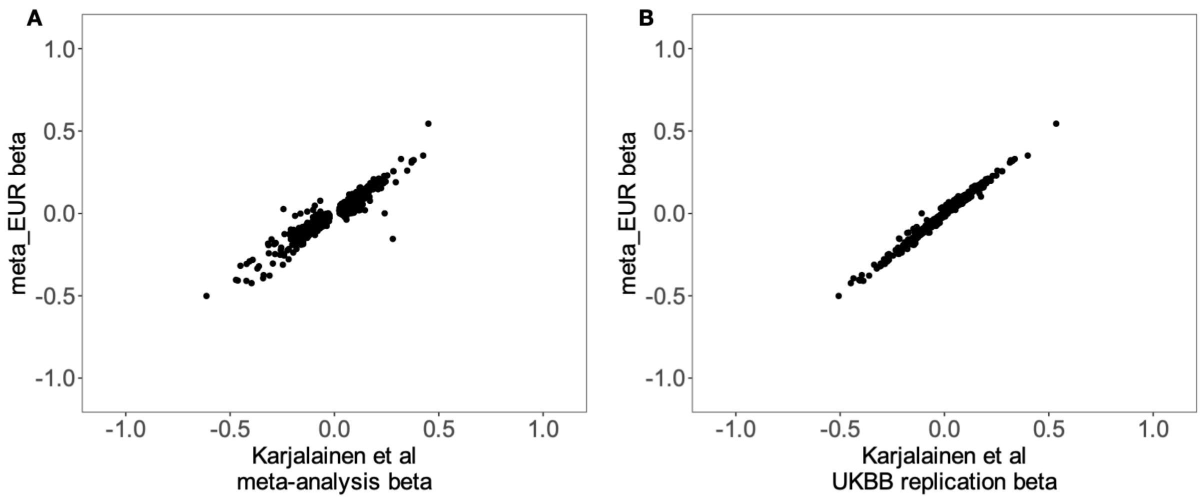


**Figure S2. Comparison of shared lead variant betas in our European-ancestry meta-analysis (meta_EUR) and results presented by Karjalainen *et al*.** (**A**) Scatter plot of GWAS lead variant effect sizes from Karjalainen *et al* main analysis (n = 137k, 33 cohorts) and our meta_EUR. (**B**) GWAS lead variant effect sizes from Karjalainen *et al* UK biobank replication (n = 100k) and our meta_EUR meta-analysis. Even though Karjalainen *et al* included 3,701 samples from the Estonian Biobank, these were older samples profiled in 2011- 2012 that were excluded from our meta-analysis due to significant batch effects. Thus, there is no sample overlap between our meta-analysis and the primary analysis conducted by Karjalainen *et al* (panel **A**). The ~100,000 UK Biobank samples used for replication by Karjalainen *et al* were also part of our meta-analysis, explaining the extremely high concordance in GWAS effect sizes.

medRxiv preprint doi: https://doi.org/10.1101/2024.10.15.24315557; this version posted April 12, 2025. The copyright holder for this preprint (which was not certified by peer review) is the author/funder, who has granted medRxiv a license to display the preprint in perpetuity.
It is made available under a CC-BY 4.0 International license .

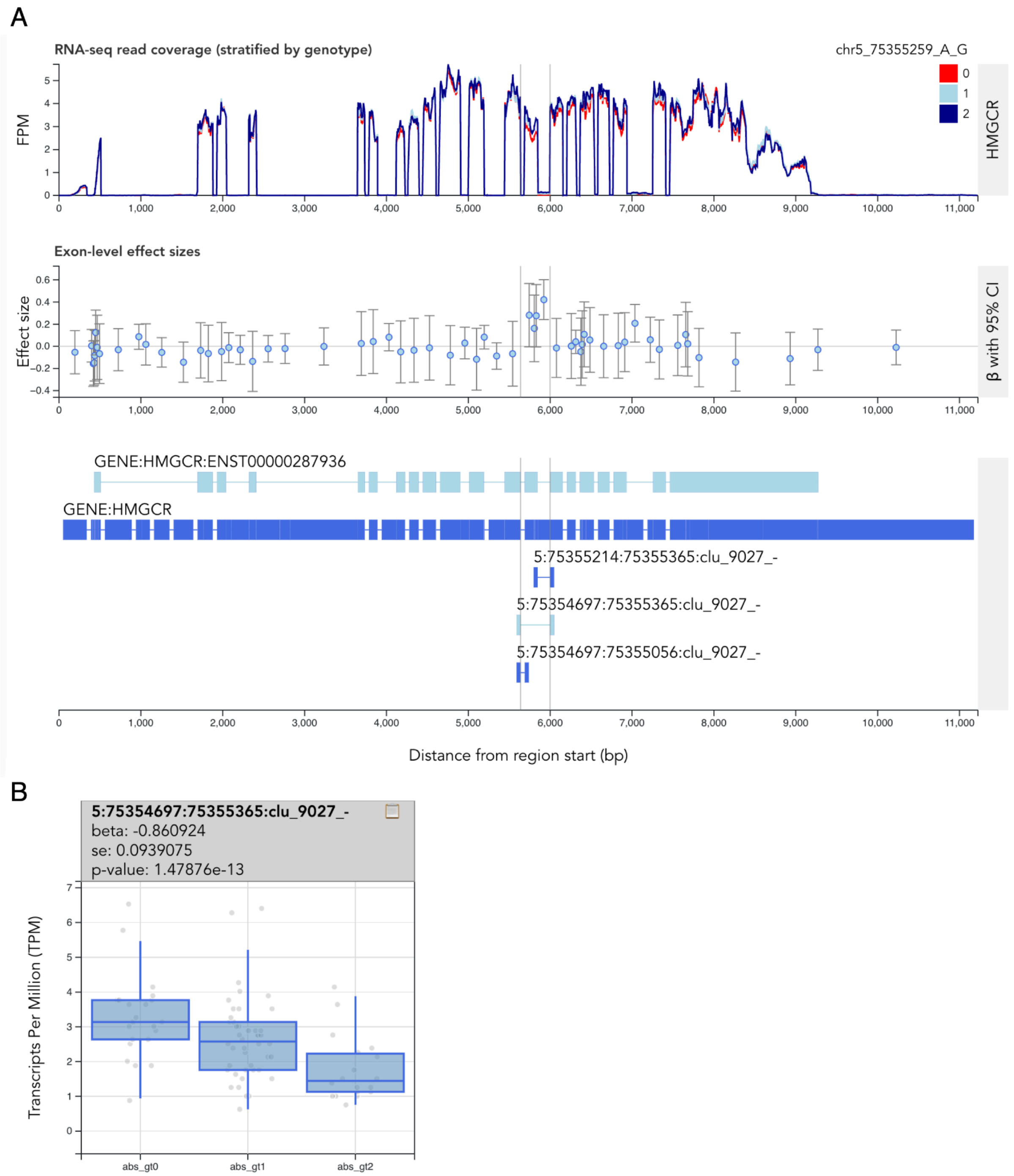


**Figure S3**. ***HMGCR* sQTL signal in the Alasoo_2018 dataset.** (**A**) RNA-seq read coverage across the HMGCR gene stratified by the genotype of the lead sQTL variant (5-75355259-A-G). (**B**) Usage of the exon13-skipping splice junction stratified by the genotype of the lead sQTL variant. Interactive visualisation available from here.

medRxiv preprint doi: https://doi.org/10.1101/2024.10.15.24315557; this version posted April 12, 2025. The copyright holder for this preprint
(which was not certified by peer review) is the author/funder, who has granted medRxiv a license to display the preprint in perpetuity.
It is made available under a CC-BY 4.0 International license .

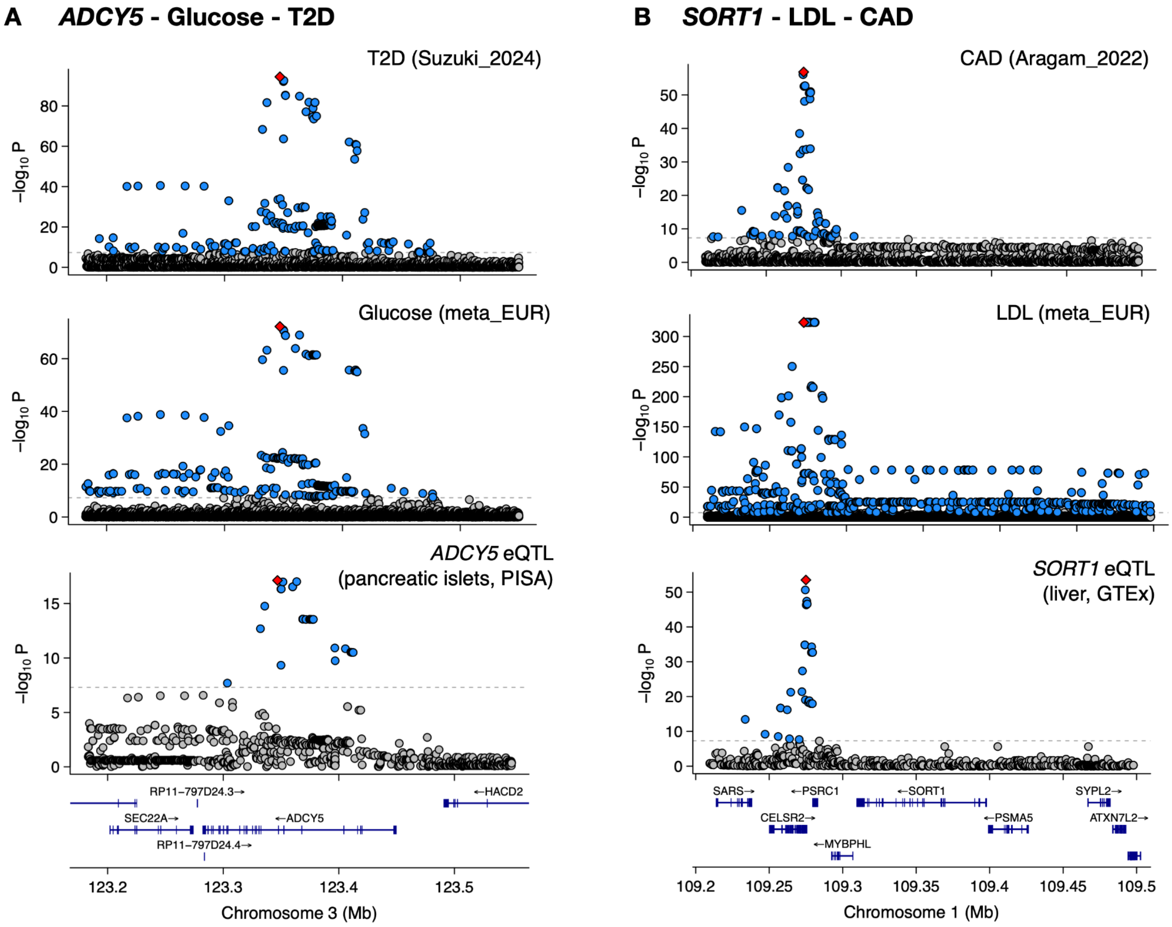


**Figure S4. Examples of colocalising eQTL - metabolic trait - disease triplets.** (**A**) Colocalisation between *ADCY5* eQTL in pancreatic islets, plasma glucose and T2D GWAS. (**B**) Colocalisation between SORT1 eQTL in the liver, plasma LDL cholesterol and CAD.

medRxiv preprint doi: https://doi.org/10.1101/2024.10.15.24315557; this version posted April 12, 2025. The copyright holder for this preprint (which was not certified by peer review) is the author/funder, who has granted medRxiv a license to display the preprint in perpetuity.
It is made available under a CC-BY 4.0 International license .

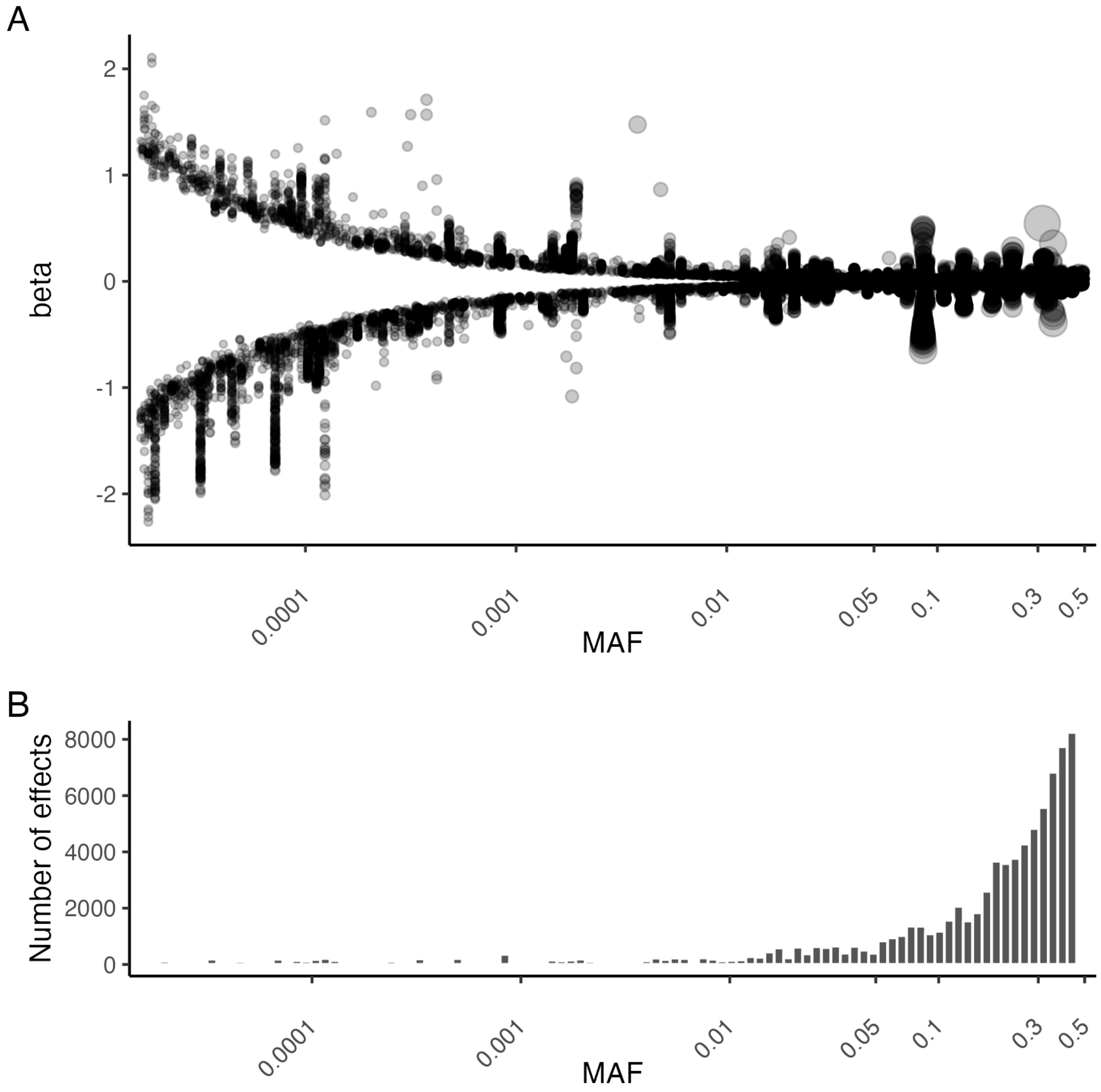


**Figure S5**. **Detected metabolite trait associations with low-frequency variants.** (**A**) Relationship between the lead variant minor allele frequency (MAF) and effect size (beta). Each dot signifies the lead variant (+/- 1Mb window) from each locus-trait pair (meta_EUR). The size of each dot has been scaled by -$\log_{10}$ p-value. (**B**) Number of detected significant associations in relation to the lead variant MAF in meta_EUR analysis.

medRxiv preprint doi: https://doi.org/10.1101/2024.10.15.24315557; this version posted April 12, 2025. The copyright holder for this preprint (which was not certified by peer review) is the author/funder, who has granted medRxiv a license to display the preprint in perpetuity.
It is made available under a CC-BY 4.0 International license .

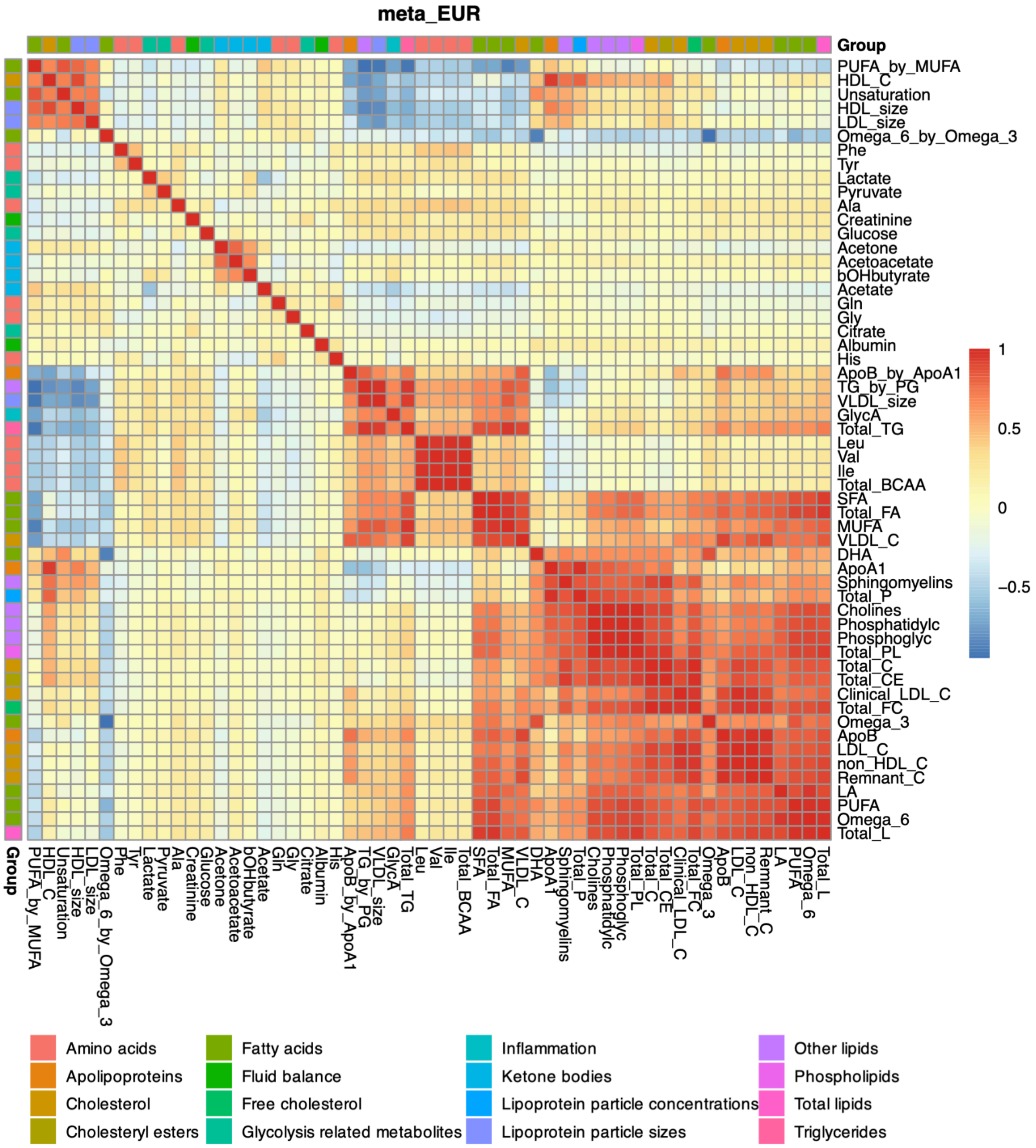


**Figure S6. Heatmap of pairwise genetic correlations between metabolic traits in the meta_EUR dataset.** The heatmap shows a representative subset of 56 metabolic traits from the main metabolic classes. The complete genetic correlation matrix for all 249 metabolic traits is presented in Table S7.

medRxiv preprint doi: https://doi.org/10.1101/2024.10.15.24315557; this version posted April 12, 2025. The copyright holder for this preprint (which was not certified by peer review) is the author/funder, who has granted medRxiv a license to display the preprint in perpetuity.
It is made available under a CC-BY 4.0 International license .

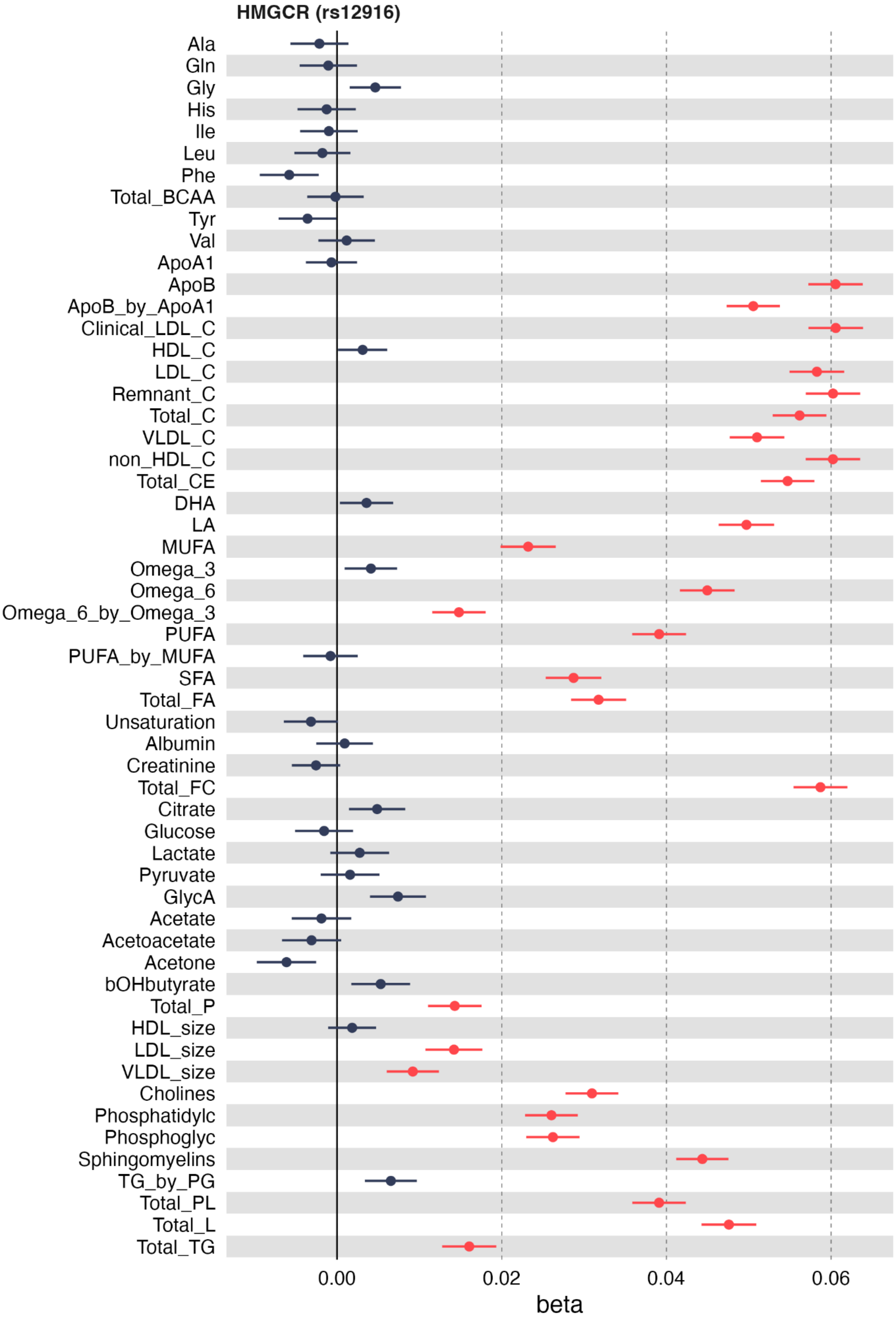


**Figure S7.** Pleiotropic effects of the *HMGCR* locus lead variant rs12916 on many lipid-related metabolites. The forest plot shows a representative subset of 56 metabolic traits from the main metabolic classes.

medRxiv preprint doi: https://doi.org/10.1101/2024.10.15.24315557; this version posted April 12, 2025. The copyright holder for this preprint (which was not certified by peer review) is the author/funder, who has granted medRxiv a license to display the preprint in perpetuity.
It is made available under a CC-BY 4.0 International license .

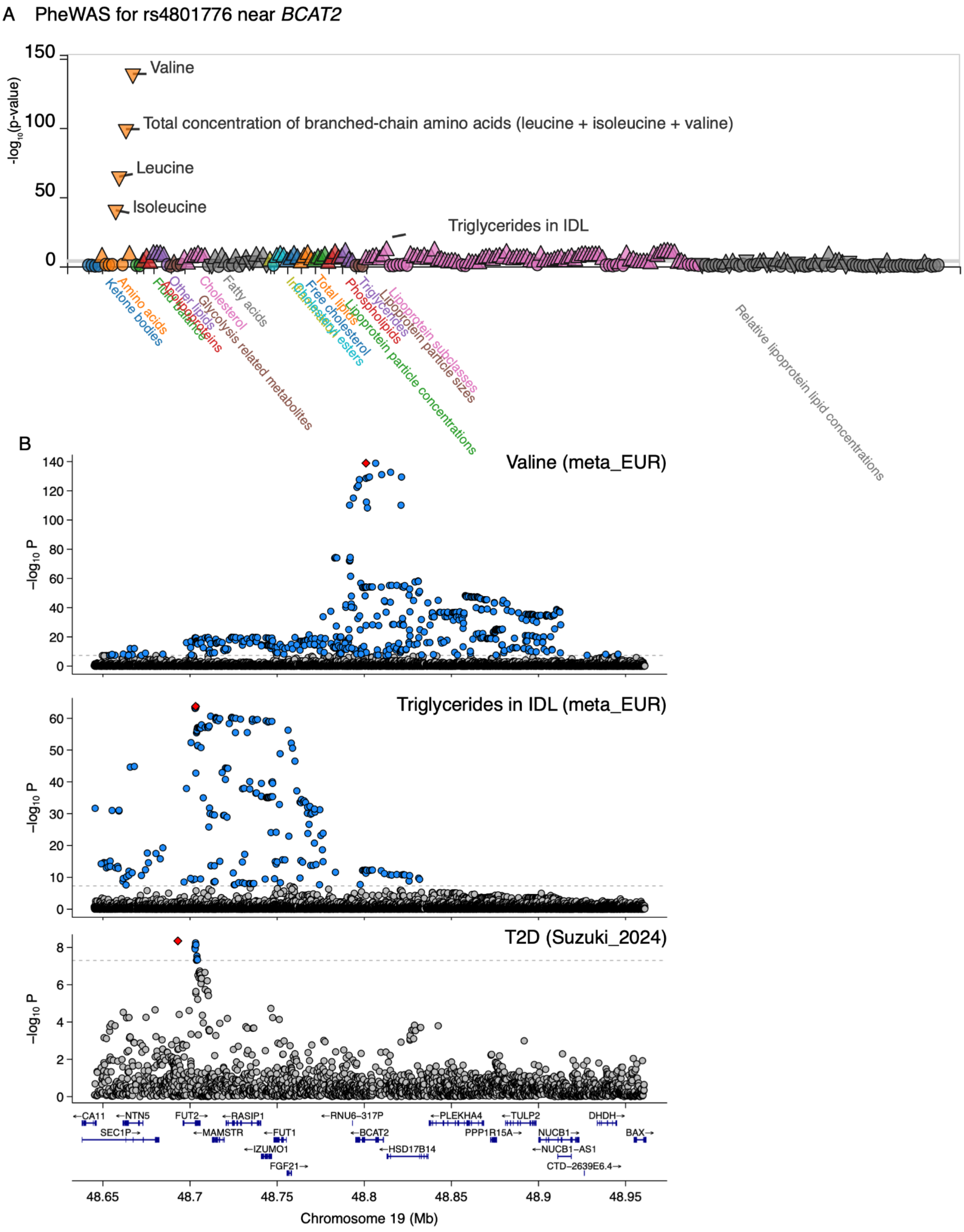


**Figure S8. Association signals for BCAAs, lipoprotein traits and T2D near *BCAT2*.** (**A**) PheWAS plot for the valine lead variant (rs4801776) in the intron of *BCAT2*. In addition to very strong associations with all three branched-chain amino acids, we also see genome-wide significant associations with various lipid traits. (**B**) Regional association plots for Valine, Triglycerides in IDL and T2D in the *BCAT2* region. The association between rs4801776 and lipid traits seems to be driven by an independent lipid signal near the *FUT2* gene that has low LD ($r^2$ = 0.08) with the valine lead variant.

medRxiv preprint doi: https://doi.org/10.1101/2024.10.15.24315557; this version posted April 12, 2025. The copyright holder for this preprint (which was not certified by peer review) is the author/funder, who has granted medRxiv a license to display the preprint in perpetuity.
It is made available under a CC-BY 4.0 International license .

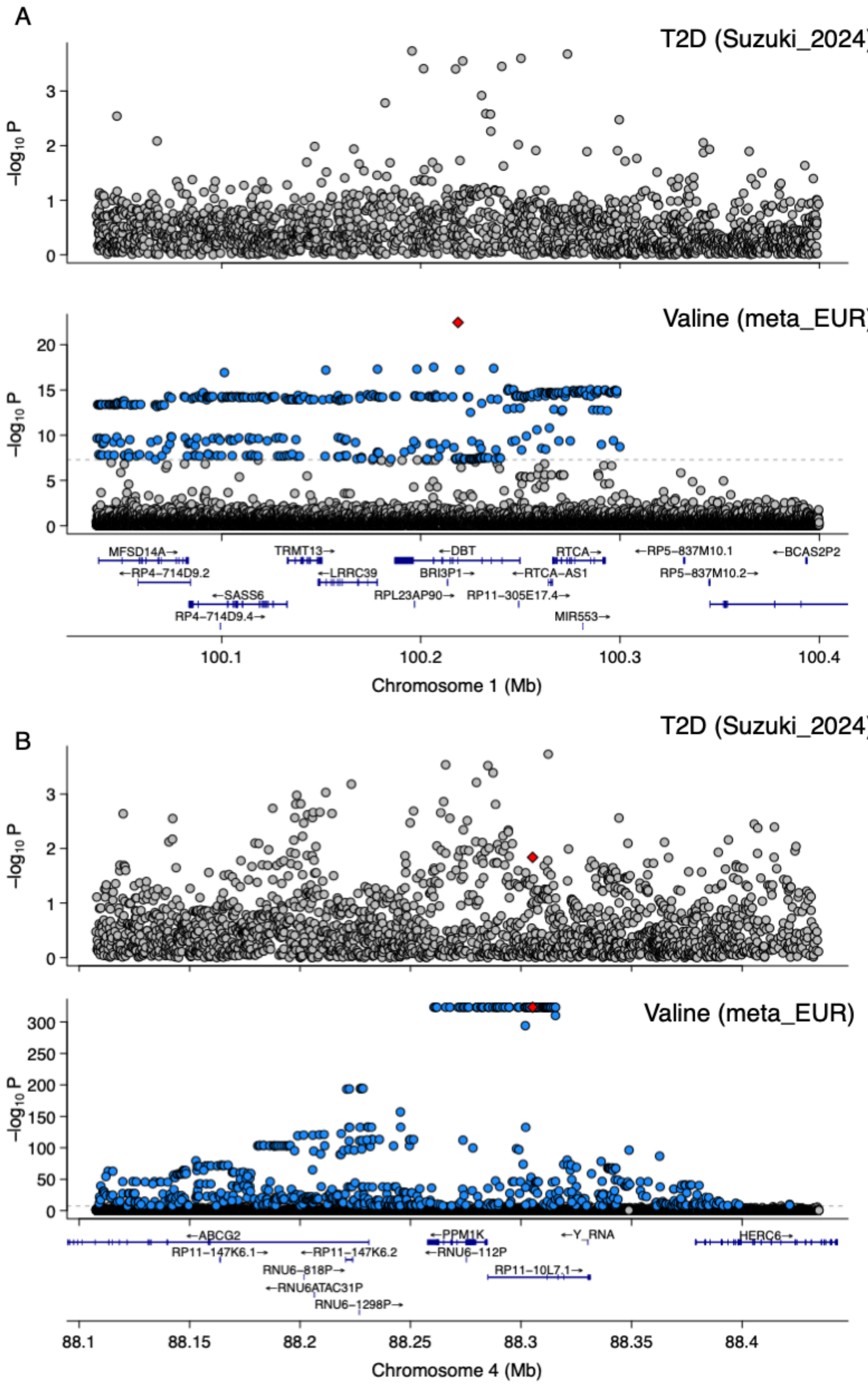


**Figure S9.** Regional association plots for T2D and valine in the *cis* regions of (**A**) *DBT* and (**B**) *PPM1K*.
